# The Effects of Bariatric Surgery and Gastrectomy on the Absorption of Drugs, Vitamins, and Mineral Elements

**DOI:** 10.3390/pharmaceutics13122111

**Published:** 2021-12-07

**Authors:** Miłosz Miedziaszczyk, Patrycja Ciabach, Edyta Szałek

**Affiliations:** 1 Department of Nephrology, Transplantology and Internal Diseases, Poznań University of Medical Sciences, 60-355 Poznań, Poland; 2 Department of Clinical Pharmacy and Biopharmacy, Poznań University of Medical Sciences, 61-861 Poznań, Poland; ciabachpatrycja@gmail.com (P.C.); szalekedyta@wp.pl (E.S.)

**Keywords:** bariatric surgery, gastrectomy, pharmacokinetics

## Abstract

Bariatric surgery, which is an effective treatment for obesity, and gastrectomy, which is the primary treatment method for gastric cancer, alter the anatomy and physiology of the digestive system. Weight loss and changes in the gastrointestinal tract may affect the pharmacokinetic parameters of oral medications. Both bariatric and cancer patients use drugs chronically or temporarily. It is important to know how surgery affects their pharmacokinetics to ensure an effective and safe therapy. The Cochrane, PubMed, and Scopus databases were searched independently by two authors. The search strategy included controlled vocabulary and keywords. Studies show that bariatric surgery and gastrectomy most often reduce the time to maximum plasma concentration (*t_max_*) and decrease the maximum plasma concentration (C_max_) in comparison with the values of these parameters measured in healthy volunteers. Vitamin and mineral deficiencies are also observed. The effect depends on the type of surgery and the properties of the drug. It is recommended to use the drugs that have been tested on these groups of patients as it is possible to monitor them.

## 1. Introduction

**Figure 1:**
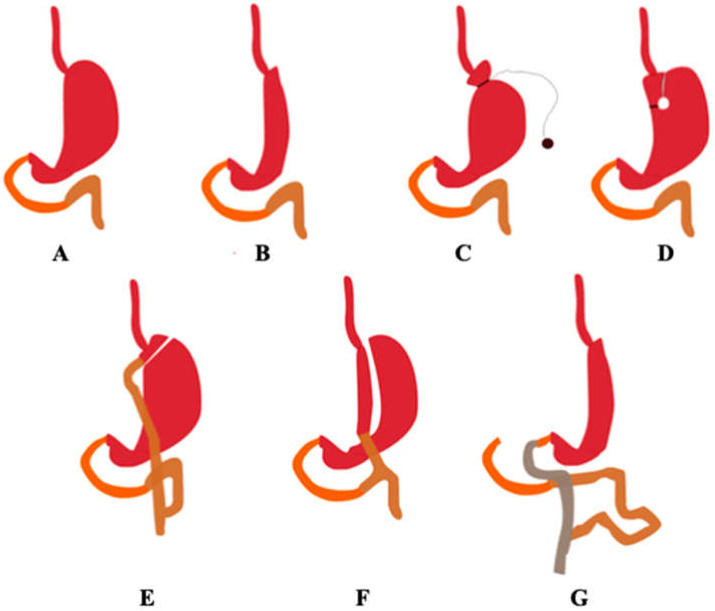
Selected types of bariatric procedures. The organs are marked with the following colours: duodenum—orange, the jejunum—brown, and the ileum—grey. (**A**) Correct anatomy, (**B**) sleeve gastrectomy, (**C**) adjustable gastric banding, (**D**) vertical banded gastroplasty, (**E**) Roux-en-Y gastric bypass, (**F**) mini gastric bypass, (**G**) biliopancreatic diversion with duodenal switch. (Authors’ original design).

Bariatric surgery is an increasingly common obesity treatment applied to patients when other treatments have not been successful. It has proved to successfully reduce the weight of patients with a body mass index (BMI) greater than 40 kg/m^2^ or 35 kg/m ^2^ in the presence of comorbidities (type 2 diabetes or cardiovascular diseases). According to the World Health Organization (WHO), in 2016 33% of adults were overweight or obese [1]. Bariatric procedures are classified as restrictive, malabsorptive, or restrictive and malabsorptive. Restrictive procedures reduce the amount of food that can be stored in the stomach but do not interfere with normal digestion, often resulting in a small gastric pouch with a narrow mouth. Examples of restrictive procedures are: (laparoscopic) sleeve gastrectomy (L)SG, (laparoscopic) adjustable gastric banding (L)AGB, and vertical banded gastroplasty (VBG). Malabsorptive procedures such as biliopancreatic diversion (BPD) consist of the shortening of the gastrointestinal tract to limit the possible extent of absorption. The following procedures combine both categories: Roux-en-Y gastric bypass (RYGB), mini gastric bypass (MGB), biliopancreatic diversion with duodenal switch (BPD-DS), single anastomosis duodeno-ileal bypass (SADI), and single anastomosis gastric-ileal bypass (SAGI) [1,2]. Figure 1 shows selected types of the abovementioned bariatric procedures.

The type of bariatric surgery affects changes in the body functions. Although the exact mechanisms of action are unknown, the effects of some bariatric procedures appear to be purely anatomical and can cause significant weight loss without significantly changing metabolic pathways. Other treatments change the anatomy of the digestive tract in a way that changes certain physiological parameters. These treatments decrease orexigenicity and increase the count of anorexogenic hormones, so physical hunger is suppressed despite progressive weight loss [3]. RYGB is the most common type of bariatric surgery [4]. It consists of creating a gastric sac with a capacity of 20–30 mL by sewing the upper part of the stomach and then restoring the continuity of the gastrointestinal tract by creating a Roux-en-Y branch of the gastrointestinal tract in the jejunum, which requires gastrointestinal and gastrojejunal anastomosis. This surgical procedure results in a weight loss of 57–67%. Weight loss is caused by both diet restriction and decreased absorption due to short-circuiting and hormonal changes [5]. In SG, 70–80% of the outer stomach is removed from the body and only a narrow gastric tube is left. While SG promotes some reduction in consumption, it also involves metabolic mechanisms of action, including increases in PYY and GLP-1, as well as an increased feeling of fullness. It also causes a permanent decrease in ghrelin levels as a result of resection of the cell mass responsible for its secretion [3]. SG results in a weight loss of 55–65% [5]. In 2016, SG was the most common primary surgery in the world (54%), followed by RYGBP (30%) [6]. Laparoscopic adjustable gastric banding (LAGB) was the most common technique of bariatric surgery for several years until its indications gradually decreased in favor of other interventions. LAGB consists of wrapping the upper part of the stomach with an adjustable band. Subcutaneous injection of physiological serum through a small port enables adjustment of the band. This intervention is purely restrictive and leads patients to change their eating behavior by inducing early satiety. LAGB allows patients to lose 40–54% of their excess weight. However, the effectiveness of this procedure declines over time as patients adjust their eating habits. Biliopancreatic diversion with duodenal switch (BPD/DS) differs from RYGB in the size of the stomach (a small 20 mL bag for RYGB; a longitudinal 150 mL bag for BPD/DS) and, more importantly, the resulting common digestive canal (100 cm for BPD/DS; about 400 cm for RYGB), which makes BPD/DS a much larger component of malabsorption. Due to many malabsorption disorders, this procedure is applied to a very small percentage of patients undergoing bariatric surgery [5].

Anatomical and physiological changes in the gastrointestinal tract occurring after bariatric surgery may change various factors and result in reduced bioavailability of drugs. The absorption of a drug strongly depends on its physicochemical properties (solubility, lipophilicity, particle size and polarity) and the physiology of the gastrointestinal tract. Bariatric surgery procedures result in bypassing part of the intestine which is rich in metabolising enzymes. This may affect the oral bioavailability of some drugs. After absorption, drugs undergo intestinal and hepatic metabolism, which is an important factor limiting their oral bioavailability. RYGBP bypasses the proximal part of the intestine, which is rich in metabolising enzymes. This bypass places drugs directly in the more distal part of the intestine, which is less metabolic, and thus results in higher oral bioavailability. The dominant drug-metabolising enzymes are cytochrome P450 (CYP) enzymes, the most numerous of which is the CYP3A subfamily. In addition to its extensive expression in the liver, the CYP3A subfamily is widely expressed in the duodenum and proximal jejunum. At least 50% of the drugs available on the market are metabolized by CYP3A. CYP3A enzymes have been reported to constitute 80% of the total P450 content in the proximal small intestine. In consequence, the gastrointestinal rearrangement after bariatric surgery, especially RYGBP, greatly affects the oral bioavailability of CYP3A substrates. Other enzymes found in the small intestine are CYP2C9, CYP2C19, and CYP2D6, as well as UDP-glucuronosyltransferases (UGTs). Drugs penetrate the intestinal mucosa by passive diffusion or active transport, depending on their solubility and lipophilicity. Transport proteins found in the gastrointestinal tract facilitate active transport. Therefore, they may also affect both the absorption and the intestinal metabolism of substrate drugs. Many different drug transporters, including P-glycoprotein (P-gp), are expressed in the gastrointestinal tract. Gastrointestinal rearrangement after bariatric surgery may affect the pharmacokinetics of drugs [6]. The Biopharmaceutical Classification System (BCS) distinguishes four classes of drugs according to their permeability and solubility. This system might help to predict some of the effects of bariatric surgery on various drugs on the basis of their physicochemical properties. Insufficient knowledge on this subject causes pharmacological problems in patients after bariatric surgery. The aim of our review was to present changes in the pharmacokinetics and pharmacodynamics of selected drug groups in patients after bariatric surgery and gastric resection [6].

Moreover, the patients classified for this surgery are often characterized by extreme obesity and it is essential to consider changes in drug pharmacokinetics typical for obesity. They result in increased lean body mass (fat body mass in obese people is also increased), accelerated gastric emptying, altered activity of enzymes involved in drug metabolism, and enhanced glomerular filtration rate [6]. Additionally, considering obese patients, a few studies have revealed metabolic differences in visceral adipose tissue (VAT) between obese and non-obese individuals, which could be the next essential aspect in pharmacotherapy of patients after gastrectomy [7,8]. Dysfunctional VAT has pro-inflammatory features and promotes cardiovascular disease and type 2 diabetes mellitus. Adipose tissue secretes adipokines, for example, which are known mediators of various metabolic processes [9]. These aspects confirm that obesity is a complex disease, also because of related health concerns. To prevent excessive weight regain and improve comorbidities (e.g., diabetes, hypertension) in bariatric patients after surgery, more frequent long-term medical follow-up visits and regular monitoring are recommended [10]. Unfortunately, approximately 20–30% of bariatric patients do not achieve successful weight outcomes, because of many factors such as food tolerance, patient knowledge, and also type of surgery [11]. We also note an interesting study by Bellia et al. It was observed that 25OHD levels were higher in metabolically healthy obese patients than in insulin-resistant obese patients. An interesting fact was highlighted: the higher the 25OHD value, the lower the risk of insulin resistance [12].

The aim of our review was to present changes in the pharmacokinetics and pharmacodynamics of selected drug groups in patients after bariatric surgery and gastric resection [13]. Each year, about one million cases of gastric cancer are diagnosed worldwide. The mortality rate in this group of patients is high, i.e., about 70–75% [6,14]. Gastric cancer patients require various forms of gastrectomy (surgical removal of part or the whole stomach) [15]. Surgery is the only treatment option [14]. Currently, three gastrectomy procedures are available: proximal gastrectomy, distal gastrectomy, and total gastrectomy. Total gastrectomy with lymph node resection is the standard procedure for treating gastric cancer. However, in the case of limited gastric cancer, it is possible to use a different gastric resection procedure [6].

**Figure 2:**
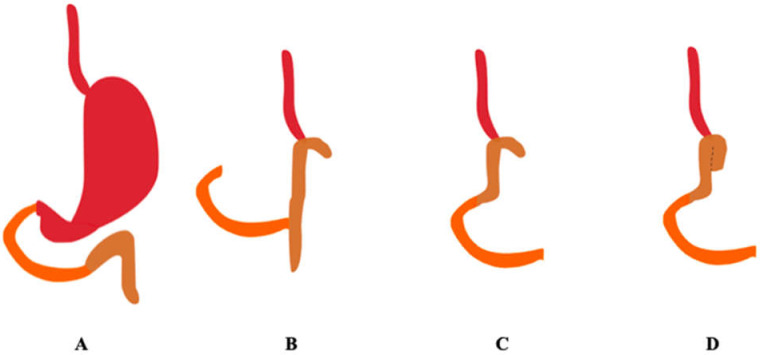
Methods of reconstruction of the gastrointestinal tract after total gastrectomy. The duodenum is marked in orange, the jejunum in brown. (**A**) Correct anatomy, (**B**) Roux-en-Y, (**C**) jejunal interposition, (**D**) jejunal interposition with pouch. (Authors’ original design).

There are several methods of reconstructing the gastrointestinal tract after total gastrectomy: Roux-en-Y reconstruction, jejunal interposition, jejunal interposition with a pouch. Roux-en-Y reconstruction consists of esophageal jejunostomy of the remaining esophagus into the jejunum and jejunojejunostomy between the initial part of the left jejunum and the first loop of the jejunum. Reconstruction can be done with or without a pouch [16]. Figure 2 shows the methods of reconstruction of the gastrointestinal tract after total gastrectomy.

**Figure 3:**
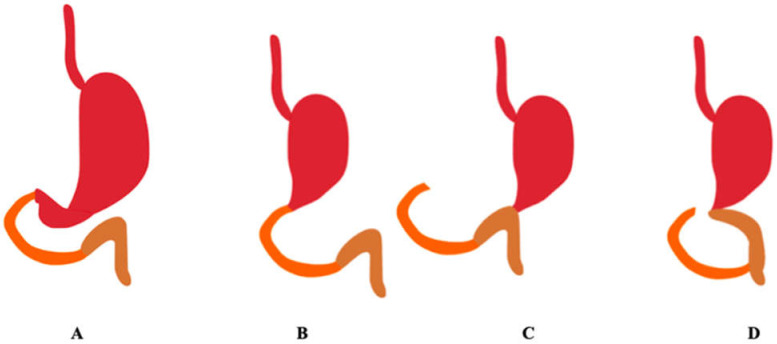
Methods of reconstruction of the gastrointestinal tract after distal gastrectomy. The duodenum is marked in orange, the jejunum in brown. (**A**) Correct anatomy, (**B**) Billroth I, (**C**) Billroth II, (**D**) Roux-en-Y. (Authors’ original design).

Reconstructions after distal gastrectomy include: Billroth I reconstruction, Billroth II reconstruction, and Roux-en-Y reconstruction. Billroth I includes a gastroduodenal anastomosis. Billroth II includes gastrojejunostomy of the remaining stomach to the first jejunal loop. Roux-en-Y includes gastrojejunostomy of the remaining stomach to an excluded jejunal limb and end-to-side jejunojejunostomy between the excluded jejunum to the first jejunal loop [10]. Figure 3 shows the methods of reconstruction of the gastrointestinal tract after distal gastrectomy.

**Figure 4:**
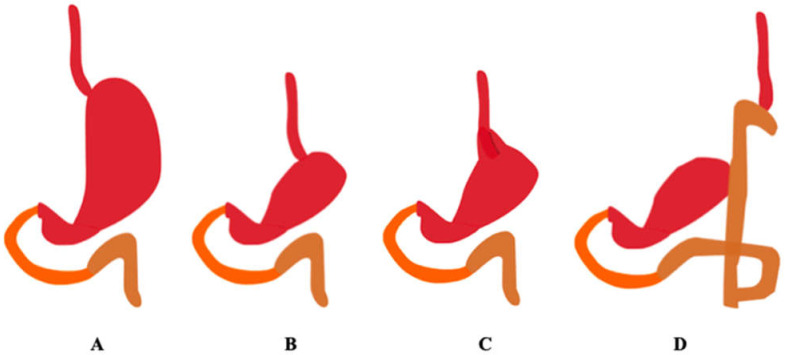
Methods of reconstruction of the gastrointestinal tract after proximal gastrectomy. The duodenum is marked in orange, the jejunum in brown. (**A**) Correct anatomy, (**B**) esophagogastrostomy, (**C**) esophagogastrostomy with fundoplication, (**D**) double tract. (Authors’ original design).

Reconstruction schemes after proximal gastric resection are currently being tested. Reconstruction after proximal gastrectomy was initially performed as direct esophagogastrostomy, but this procedure involves a high rate of gastric reflux. To prevent the occurrence of a gastric reflux, different approaches have been tested, e.g., combining esophagogastrostomy with fundoplication, jejunal interposition with and without a pouch, double tract reconstruction, and ileocolic interposition [16]. Figure 4 shows the methods of reconstruction of the gastrointestinal tract after proximal gastrectomy.

Various forms of gastrectomy may significantly change the pharmacokinetics of orally taken drugs [17]. The lack of stomach results in mechanometabolic and deficiency metabolic disorders. The former group of disorders includes postprandial syndrome and alkaline esophagitis due to regurgitation. The latter group of disorders includes anemia, osteoporosis and/or osteomalacia, and weight loss [18].

The consequences of gastric surgery, such as reduced gastric volume, reduced secretion of gastric, pancreatic and biliary juices, accelerated gastric emptying, and impaired fat absorption, may affect the pharmacokinetics of drugs [6,15]. The increase in gastric pH after gastrectomy may limit the absorption of acidic drugs [18]. Gastrectomy may change the rate and range of drug absorption by altering the time of gastric emptying into the small intestine. In consequence, the following parameters may change: area under the plasma concentration time curve (AUC), maximum drug concentration in blood (C_max_), time to maximum drug concentration in blood (*t_max_*), absorption rate constant (k_a_), bioavailability (F), and biological half-life (*t_0.5_*), which determine the therapeutic effect of the drug, its efficacy and result in treatment-induced toxicity [14]. Of course, it should be highlighted that differences between benign disease and cancer patients are relevant. A 2018 meta-analysis showed that obese patients undergoing surgery for malignancy were at increased risk of major complications, whereas obese patients undergoing surgery for benign indications were at decreased risk compared to normal weight patients [19].

## 2. Materials and Methods

The search strategy included controlled vocabulary and keywords. The Cochrane, PubMed, and Scopus databases were searched independently by two authors. The main search concept was to combine ‘gastrectomy’, ‘gastric bypass’, ‘bariatric surgery’ with related terms such as ‘pharmacokinetic’, ‘absorption’, ‘changes’, and ‘bioavailability’. The inclusion criterion was the data included in the studies related to the groups of drugs selected by the authors. Due to the small number of studies in recent years, the time criterion was not applied. Table 1 shows the steps for including articles in the review.

### 2.1. Antibiotics

Rocha et al., conducted a study on patients (*n* = 8) before and two months after the RYGB procedure to investigate changes in the pharmacokinetics of amoxicillin (AMX) [4]. AMX is the most common antibiotic, used since the 1970s, with good absorption (85–90%), especially in the duodenum and jejunum [20]. The drug has a non-linear absorption profile, so it means that the process rate is saturable [21]. Rocha et al., conducted research on obese subjects who received a single dose of amoxicillin in a 500 mg capsule. After the surgery, the mean weight loss was 17.03 ± 5.51 kg, and the mean body mass index (BMI) decreased from 46.21 ± 2.82 to 38.82 ± 3.32 kg/m^2^. The mean amoxicillin area under the plasma concentration versus time curve from time zero to the time of the last quantifiable concentration (AUC_0–last_) increased significantly (2.03 vs. 7.21 μg∙h/mL; *p* = 0.0224); the peak plasma concentration (C_max_) also increased significantly (0.62 vs. 1.77 μg/mL; *p* = 0.0279) after bariatric surgery. There was no correlation between amoxicillin absorption, BMI, and weight loss percentage. The changes observed in the pharmacokinetics of amoxicillin suggest that the obese subjects enrolled in this study had significant increases in the systemic amoxicillin exposure after the RYGB surgery. However, despite this increasee, this exposure was lower than that of the non-obese volunteers, whose AUC_0–last_ values ranged from 12.44 to 12.05 μg∙h/mL, whereas their C_max_ ranged from 4.94 to 5.31 μg/mL after a single oral administration of 500 mg amoxicillin capsules [4]. This may be related to body mass. Mellon et al., observed that the amoxicillin C_max_ decreased significantly with weight. Considering the target PK/PD value for beta-lactams fT > MIC ≥ 40%, the standard dosage of co-amoxiclav (1000/125 mg every 8 h) should be efficacious for obese adults [22], but Soares et al., suggested that amoxicillin treatment would fail if standard therapeutic regimens were applied because of a significantly higher volume of distribution in this group of patients [23].

In another study, the bioavailability of an oral AMX tablet and suspension was tested on patients who had undergone an RYGB surgery 3 months to 10 years before. The patients received an 875 mg AMX tablet or 800 mg AMX suspension. Twenty people with a body mass index of 29.88 ± 4.36 kg/m ^2^ were qualified for the study. The C_max_ of AMX in the plasma of tablets and suspensions (normalized to 875 mg) was 7.42 ± 2.99 mg/L and 8.73 ± 3.26 mg/L (90% CI = 70.71–99.11), and the area under the plasma concentration versus time curve from time zero to infinity (AUC_0–∞_) was 23.10 ± 7.41 mg⋅h/L and 27.59 ± 8.32 mg⋅h/L, respectively (Cl = 71.25–97.32). The values of these parameters were compared with the results noted in healthy subjects, as described in available literature. The healthy subjects received 875 mg AMX tablets (alone or in combination with clavulanic acid). The AUC_0–∞_ and C_max_ values increased from 43.80 to 51.29 mg⋅h/L and from 12.13 to 15.30 mg/L [24].

Padwal et al., conducted a study on the pharmacokinetics of azithromycin, which is a macrolide antibiotic with a broad spectrum of activity against various aerobic and anaerobic bacteria [25]. Azithromycin is preferentially absorbed in the duodenum and upper jejunum. The oral bioavailability of azithromycin in healthy subjects amounts to about 37% [26]. A total of 14 women who were at least 3 months post RYGB surgery and 14 healthy women (the control group) with matched body mass index (BMI) (mean age 44 years and BMI 36.4 kg/m^2^) were administered a single dose of two 250 mg azithromycin tablets. The AUC_0–24_ of the patients who had undergone the RYGB procedure was reduced by 32% (1.41 vs. 2.07 mg⋅h/L; *p* = 0.008), whereas the dose-normalized AUC_0–24_ was reduced by 33% (0.27 vs. 0.40 kg⋅h/L; *p* = 0.009). The azithromycin C_max_ of the patients after the RYGB surgery amounted to 0.260 mg/L, as compared with 0.363 mg/L in the control group (*p* = 0.08) and it was reached after 2.14 h and 2.36 h (*p* = 0.75), respectively. These results show that there is a possibility of early treatment failure. Therefore, modified dosage and closer clinical monitoring of gastric bypass patients receiving azithromycin should be considered [27]. The PK/PD relationship for azithromycin is AUC/MIC. Therefore, a lower AUC in patients after gastrectomy may cause treatment failure.

Ciprofloxacin is a fluorinated quinolone antibiotic with high activity against a wide spectrum of Gram-positive and Gram-negative bacteria. Clinical trials with an orally administered ciprofloxacin preparation proved the effectiveness of this drug in the treatment of urinary tract infections, sexually transmitted infections, skin, bone and joint infections, prostatitis, typhoid fever, gastrointestinal infections, lower respiratory tract infections, anthrax, plague, and salmonellosis. Significant quantities of ciprofloxacin are absorbed after its oral administration. The drug is mainly absorbed in the upper part of the intestinal tract (duodenum, jejunum). The absolute bioavailability is about 70% [28]. Rivas et al., conducted a study on the pharmacokinetics of ciprofloxacin after its single administration to patients after an RYGB surgery. The study involved overweight and obese patients aged 18–60 years. The assessment was performed once in the control group and three times in the group of overweight and obese patients (first before the surgery and then one and six months after the surgery). The subjects received a single oral dose of 500 mg of ciprofloxacin at each visit. Taking the postoperative change in body weight into account, the parameters were adjusted according to the dose (mg)/body weight (kg). The ciprofloxacin C_max_ decreased significantly one month after the surgery (1840.9 ± 485.2 vs. 1459.6 ± 354.8 ng/mL; *p* = 0.032), but not after six months (1840.9 ± 485.2 vs. 1589.6 ± 32.8 ng/mL; *p* = 0.116). The C_max_ measured after sixth months was lower than the C_max_ in the control group (2160.4 ± 408.6 vs. 1589.6 ± 321.8 ng/mL; *p* < 0.001). The AUC_0–∞_ of ciprofloxacin decreased significantly one month after the surgery (9141.3 ± 1774.0 vs. 7581.4 ± 1511.1 h⋅ng/mL; *p* = 0.014), but not after six months (9141.3 ± 1774.0 vs. 9067.6 ± 3880.2 h⋅ng/mL; *p* = 0.947). Six months after surgery, the C_max_ and AUC_0–∞_ values were lower than in the control group (1589.6 ± 32.8 vs. 2160.8 ± 408.6 ng/mL; *p* < 0.001 and 9067.6 ± 3880.2 vs. 9737.2 ± 2717.6 h∙ng/mL; *p* = 0.564, respectively). The C_max_/MIC for fluoroquinolones should be greater than 10, although some studies suggest that the AUC24/MIC ratio is more accurate for this group of chemotherapeutic agents. The AUC24/MIC value for fluoroquinolones depends on the causative pathogens (G(+) > 40, G(−) = 100–125) [29]. In conclusion, by the sixth month, the effect on the C_max_ and AUC_0–∞_ had disappeared due to weight loss. There is no need to modify the doses of ciprofloxacin in these patients [30].

As results from the abovementioned studies, the values of pharmacokinetic parameters may be influenced by the time after bariatric surgery. Another conclusion concerns the form of the drug. It is important to note that the suspension and the tablet affect the pharmacokinetic parameters differently in patients after RYGB surgery.

### 2.2. Analgesic Drugs

Acetaminophen is an analgesic drug of choice for patients after gastrectomy, even in oral formulations. This drug is mainly absorbed by passive transport in the small intestine [31]. The oral route of administration of acetaminophen increases the risk of postoperative nausea and vomiting, as compared with the intravenous route, but the efficacy of both routes of administration is comparable [32]. Porat et al., conducted a clinical, crossover study on the pharmacokinetics of paracetamol in obese patients enrolled for LSG. The patients received randomly 500 mg of paracetamol in a caplet or in syrup. The other form of the drug was administered after 1–2 weeks. The study was repeated 4–6 months after the surgery. The mean weight loss was 26 kg. The researchers observed that the AUC and C_max_ were higher after these few weeks than before the surgery. The bioavailability of paracetamol increased twice and it was higher when administered as a liquid. The *t*_1/2_ was longer after LSG. The changes in the pharmacokinetic parameters were associated with the patients’ loss of weight. The bioavailability of acetaminophen in obese patients was much lower [17]. This may have been caused by an increase in the metabolic pathways (including glucuronidation) in these patients [33]. In addition, LSG accelerated gastric emptying, so the *t_max_* in the syrup group was shorter after the surgery [17].

The pharmacokinetics of paracetamol were also investigated in patients after total gastrectomy. Szałek et al., conducted a study comparing the pharmacokinetic parameters after the administration of two generic products. A group of 30 people after gastrectomy with Roux-en-Y reconstruction was divided into two groups. The participants received two tablets containing 500 mg of paracetamol each. The C_max_ and AUC in both groups were lower than in healthy subjects. The *t_max_* and *t*_1/2_ were similar to the values of these parameters measured in the volunteers without gastrectomy. The results suggest that total gastrectomy reduces the absorption of this drug [34].

Tramadol is another analgesic drug. It is indicated for the treatment of chronic and postoperative pain, renal and biliary colic, and trauma. Tramadol is a weak opioid. It is often used in combination with paracetamol at a dose of 37.5 mg tramadol and 325 mg paracetamol. This combination is in the form of conventional or effervescent tablets [35]. When administered orally, its absorption in the upper small intestine amounts to 95–100% [36]. The pharmacokinetics of these two forms of paracetamol and tramadol were investigated in patients after gastrectomy with Roux-en-Y reconstruction. A total of 26 patients were divided into two groups. The first group received two film-coated tablets, whereas the other group received two effervescent tablets. Each tablet contained 37.5 mg of tramadol and 325 mg of paracetamol. The C_max_ of paracetamol administered orally as a conventional tablet and the C_max_ of tramadol administered orally as an effervescent tablet were significantly lower than in healthy subjects. The *t_max_* of paracetamol administered in the form of effervescent tablets to the gastrectomy patients and the *t_max_* of tramadol in both groups were shorter. According to the researchers, this may have been caused by the shorter gastric emptying time. According to Szałek et al., conventional tablets are a better choice for patients after gastrectomy [35].

Ketoprofen belongs to the group of non-steroidal anti-inflammatory drugs. The indications for the use of this drug are postoperative pain, cancer, and rheumatoid arthritis. Ketoprofen is absorbed by passive diffusion in the stomach. It occurs in an undissociated form. Porażka et al., investigated the effect of gastrectomy on the pharmacokinetics of ketoprofen administered orally to two groups of patients. One group (*n* = 15) consisted of patients after total stomach resection, whereas the other group (*n* = 5) included patients after partial resection. All the participants received one film-coated tablet containing 100 mg of ketoprofen. The C_max_ of the patients after total stomach resection was significantly lower than in the group of the patients after partial resection. According to the researchers, this may have been caused by reduced tablet disintegration and slower mixing of the gastric contents due to the smaller size of the stomach. Faster gastric emptying, higher gastric pH and a smaller absorption area may result in lower C_max_ and *t_max_* in gastrectomy patients. The patients had higher V_d_ (volume of distribution), most likely due to hypoalbuminaemia, which is a common symptom of gastric cancer. Gastrectomy patients may require higher doses of ketoprofen for effective pain relief [6].

Morphine is the most commonly used opioid to treat moderate to severe pain. After oral administration of the drug, its absorption from the gastrointestinal tract amounts to almost 100%. Morphine is a substrate of P-glycoprotein [37]. It is absorbed mainly in the upper part of the small intestine and, to a lesser extent, in the stomach. The absolute bioavailability of morphine is low (20–30%) due to the first pass effect [38]. A study was conducted on 30 patients to determine the effect of RYGB on the pharmacokinetics of this drug. Each patient received an oral dose of 30 mg of liquid morphine at each of three visits (7–30 days before the surgery, 7–15 days after the surgery, and 6 months after the surgery). The *t_max_* decreased, whereas the C_max_ increased significantly. The AUC also increased. The study showed that RYGB significantly increased the rate of morphine absorption. The increase in the C_max_ and AUC may also have been caused by reduced first pass metabolism and weight loss, because non-obese patients have less glucuronidation than obese ones. According to researchers, after RYGB patients should receive lower doses of morphine in the form of a solution before the surgery to reduce the risk of side effects. Sublingual, intranasal or gingival application of fentanyl can be an alternative to immediate-release forms of morphine [39].

Post-operative pain is also treated with oxycodone. This semi-synthetic opioid is stronger than morphine [40]. It is absorbed mainly in the small intestine [41]. Szałek et al., conducted a study on the pharmacokinetics of oxycodone in patients after total gastrectomy. A total of 24 patients received prolonged-release tablets containing 10 mg of oxycodone. The mean C_max_ and systemic exposure of oxycodone in the men were higher than in the women. The *t_max_* of the patients after resection was slightly shorter than that of the healthy patients. This effect may have been caused by the shorter gastric emptying time. The C_max_ was similar in both groups. The study showed that total gastrectomy did not affect the pharmacokinetics of oxycodone [40].

### 2.3. Antidepressants

About 30–50% of patients after bariatric surgery use psychotropic drugs, mostly antidepressants [42]. It is important to determine changes in the pharmacokinetics of these drugs after surgery to ensure the safety and effectiveness of therapy [43].

Escitalopram belongs to the group of selective serotonin reuptake inhibitors (SSRIs). It increases synaptic signalling. It is used to treat major depression and generalized anxiety disorder. It is rapidly absorbed when administered orally. Marzinke et al., conducted a study on the pharmacokinetics of escitalopram in patients after Roux-en-Y gastric bypass surgery. There were four obese patients who used 10 or 20 mg escitalopram once a day. Samples were taken two weeks before the surgery and two and six weeks after the surgery. The serum levels of the drug decreased after surgery. At the third visit, the drug levels were even lower than at the second visit. Obese patients have elevated levels of C-reactive protein (CRP), which indicates current inflammation. This may result in decreased activity of CYP 450 enzymes and a higher concentration of escitalopram before the bariatric surgery. Another reason may be altered absorption after the surgery [42].

Sertraline also belongs to the SSRI group. Apart from depression, it is also used to treat social phobia, obsessive-compulsive disorders, post-traumatic stress disorder, and panic disorder [44]. It is mainly absorbed in the duodenum. Roerig et al., conducted a study on five RYGB patients (9–15 months after the surgery) and five non-surgical patients as the control group. The aim of the study was to determine changes in the pharmacokinetics of sertraline after the bariatric surgery. All participants received a single 100 mg dose of sertraline. The AUC_0–10.5_ and C_max_ were significantly lower in the postoperative group. The *t_max_* did not differ significantly between the two groups [43].

Depression is also treated with duloxetine. This drug is also used to treat anxiety disorders and neuropathic pain. Roerig et al., conducted a study to determine the effect of Roux-en-Y gastric bypass on the pharmacokinetics of duloxetine. Ten patients who had undergone RYGB 9–15 months before and 10 volunteers from a control group received a single dose of 60 mg of duloxetine. The postoperative patients had significantly lower AUC_0–∞_ and shorter *t_max_* than the control group. The differences in the C_max_ and half-life were not clinically significant. The researchers speculated that the absorption of duloxetine was reduced as a result of surgery and the loss of the absorptive surface of the duodenum [45].

Venlafaxine is a norepinephrine reuptake inhibitor. It is used to treat depression, social anxiety disorder, generalized anxiety disorder, and panic disorder. It is available as an immediate-release and extended-release drug [46]. Ten RYGB patients were enrolled in a prospective study of venlafaxine pharmacokinetics. At least one week before and 3–4 months after the surgery, the participants received one 75 mg capsule of ER venlafaxine. The AUC_0–24_, C_max_, and *t_max_* values measured before and after the RYGB did not differ significantly. According to Krieger et al., gastric bypass surgery does not significantly affect the amount and time of venlafaxine absorption [47].

Vortioxetine is a multimodal serotonin modulator used to treat depression. It acts on the 5-HT1A, 5-HT1B, 5-HT3, and 5-HT7 receptors and inhibits the reuptake of serotonin. Vandenberghe et al., presented a case report of a patient who underwent RYGB and used vortioxetine regularly. The blood level of the drug was determined 126 and 200 days before surgery. The drug was administered at a dose of 10 mg/day. The vortioxetine levels were also measured 91 days after surgery. The concentration was more than twice lower than before the surgery. The dose was increased to 20 mg/day. On days 224 and 308 after the surgery, the concentration was similar to that in the preoperative period. Researchers recommend therapeutic drug monitoring and crushing tablets or using a liquid form of the drug in the case of poor absorption [48].

### 2.4. Anticoagulant Drugs

Rivaroxaban belongs to the group of direct oral anticoagulants (DOAC). It is an inhibitor of factor Xa. It is used to prevent venous thromboembolism [49]. Rivaroxaban is absorbed in the upper gastrointestinal tract. The drug is characterized by high oral bioavailability [50]. Kröll et al., conducted a study comparing the pharmacokinetic and pharmacodynamic parameters of rivaroxaban administered one day before and three days after bariatric surgery. Six sleeve gastrectomy patients and six Roux-en-Y gastric bypass patients participated in the study. All of them received 10 mg of rivaroxaban each time. A slight increase in the AUC was observed after both types of surgery. The C_max_ was higher after SG and lower after RYGB than before the surgery. The *t_max_* increased in the patients after RYGB. According to the researchers, the bariatric surgery did not significantly affect the pharmacokinetic and pharmacodynamic parameters [49]. The influence of weight loss after bariatric surgery on the pharmacokinetics and pharmacodynamics of rivaroxaban was investigated on six post-SG and six post-RYGB patients, who received a single 10 mg dose of rivaroxaban 6–8 months after the surgery. The results were compared with the values measured before the surgery. Six months after the surgery the mean weight loss was over 34 kg. The postoperative *t_max_* was slightly longer than before the surgery. The C_max_ was lower after the RYGB surgery, whereas the C_max_ of the patients after SG was similar to the value measured before the surgery. Kröll et al., observed that weight loss and bariatric surgery did not significantly affect the pharmacokinetics and pharmacodynamics of rivaroxaban. The researchers assumed that changes in the body weight did not affect these parameters due to the high degree of plasma protein binding of this drug and its low volume of distribution [50].

Dabigatran is another drug from the DOAC group. It is used to treat and prevent venous thromboembolism and to prevent stroke and systemic embolism [51]. The drug is absorbed in the lower stomach and duodenum. Grainger et al., measured the pharmacokinetic and pharmacodynamic parameters (including C_max_) of dabigatran in patients (*n* = 9) after laparoscopic Roux-en-Y gastric bypass who regularly used this drug and compared them with the results from phase II studies. The drug concentration decreased significantly. According to the researchers, this may indicate impaired or delayed absorption [52]. Rottenstreich et al., observed that the C_max_ of dabigatran in BS patients regularly using this drug was within the expected range [53].

Similarly to rivaroxaban, apixaban is also a direct factor Xa inhibitor. It is indicated to reduce the risk of stroke and thromboprophylaxis and to treat deep vein thrombosis and pulmonary embolism. Apixaban is mainly absorbed in the small intestine. Its bioavailability amounts to about 50% [54]. The study assessing the effect of BS on the level of DOAC included nine patients who took apixaban chronically. The blood levels of this drug were measured and compared with the values for the general population. The peak apixaban level was within the expected range [55]. Bitar et al., reported a failure of apixaban anticoagulant therapy in a patient who had undergone bariatric surgery four years before. The researchers suggested that the surgery may have contributed to the subtherapeutic level of the drug [55].

Warfarin is an antagonist of vitamin K. It reduces the activity of coagulation factors (II, VII, IX, X) and therefore it is used to prevent and treat thromboembolic disorders. Its efficacy is measured with the International Normalized Ratio (INR). Warfarin is absorbed in the proximal duodenum. Steffen et al., conducted a retrospective study to collect data on warfarin dosage in patients after RYGB surgery. The results measured six months before and after the surgery were analysed. The mean weekly doses before and after the surgery differed significantly. After the surgery the dose was reduced by approximately 25%. It was necessary to apply lower doses after than before the surgery to maintain the INR level. The cause of the changes in the warfarin parameters was not fully explained. According to the researchers, they may have been caused by changes in the consumption and storage of vitamin K, as well as changes in the bacterial flora [56]. Vitamin K antagonists are believed to be a better choice than DOAC for patients after BS, as they are easy to monitor and to make dose adjustments [53].

### 2.5. Immunosuppressants

Adequate modulation of the immune system after transplantation is essential for patient survival and prevents rejection of the transplanted organ. A three-drug regimen of corticosteroid, mycophenolate mofetil, and tacrolimus is the most common. Cyclosporine and sirolimus are also used in immunosuppressive therapy [57]. Only reliable absorption from the gastrointestinal tract can ensure adequate exposure and efficacy of these drugs [58]. Mycophenolate mofetil is mainly absorbed in the proximal gastrointestinal tract. It is a prodrug activated during first pass metabolism. Tacrolimus is available as an immediate-release (IR-TAC) and extended-release drug (ER-TAC). The bioavailability of both forms is relatively low. IR-TAC is absorbed from the duodenum to the colon, whereas ER-TAC is absorbed more distally in the gastrointestinal tract [59]. Ciclosporin is a drug with a narrow therapeutic index. As the absorption of the drug is highly variable, it is important to monitor its concentration. Absorption is incomplete and slow. It occurs mainly in the upper intestine [60]. Sirolimus, like tacrolimus, is mainly absorbed in the duodenum [61]. Its bioavailability is low (approximately 25%) [62].

Chan et al., conducted a prospective study on changes in the pharmacokinetics of immunosuppressants. Twelve patients with end-stage renal disease were involved. The pharmacokinetic parameters after administration of a single dose of tacrolimus, extended-release tacrolimus, mycophenolate mofetil, and enteric-coated mycophenolate sodium (EC-MPS) were measured two months before and 9–12 months after LSG. First, 3 mg of IR-TAC and 720 mg of EC-MPS were administered orally. Two weeks later, the patients received orally 6 mg of ER-TAC and 1000 mg of mycophenolate mofetil (MFF). The median excess body weight loss (EBWL) was 26.5 kg. The study revealed a significant increase in the exposure to all four drugs after LSG. The exposure to tacrolimus was reduced in obese patients. After weight loss, the AUC and C_max_ of both tacrolimus forms were significantly higher. The *t_max_* and *t*_1/2_ did not change significantly. The researchers speculated that the increased exposure to tacrolimus was caused by a decrease in the P-glycoprotein expression. They also suggested that accelerated gastric emptying after the surgery resulted in earlier delivery of tacrolimus to the proximal intestine, which also increased exposure to this drug. After LSG, the apparent total plasma clearance (Cl/F) of both forms of mycophenolate mofetil decreased by about 60%. According to the researchers, the UGT activity decreased due to weight loss. The MFF *t*_1/2_ was several times longer after LSG. The *t_max_* of EC-MPS decreased by over 30%. It is recommended to monitor immunosuppression in patients after LSG [63].

The pharmacokinetics of immunosuppressants were also investigated on patients after Roux-en-Y gastric bypass (approximately 2 months to 8 years after surgery). The study involved four dialysis patients and two kidney transplant patients. All the dialysis patients received orally one dose of sirolimus (6 mg), two doses of MMF (1000 mg), and two doses of tacrolimus (4 mg) within 24 h. The patients after transplantation remained on their maintenance regimen. The pharmacokinetic parameters were compared with the results of other studies. The comparison of the AUC:dose ratio of the patients under study with the ratio of healthy volunteers showed that after gastric bypass surgery, a higher dose of sirolimus was necessary to achieve the same exposure as in healthy subjects. The patients with a gastric bypass had much lower AUC_0–12_ and AUC_0–∞_ than the healthy volunteers. As with sirolimus, the study showed that patients after bariatric surgery require a higher dose of tacrolimus. According to the researchers, this was due to reduced absorption in the small intestine. The C_max_ and mean AUC_0–12_ MPA of the patients under study were lower, which may have been caused by the reduced absorption area [61].

Chen et al., presented a case study of a patient after total gastrectomy with Roux-en-Y reconstruction with end-stage renal disease. The gastrectomy was done 5 years earlier. The patient had pharmacokinetic tests before transplantation to select the most appropriate immunosuppressive regimen. Five different treatment regimens, including EC-MPS, MMF, cyclosporine, tacrolimus, and sirolimus were tested. The regimens were tested sequentially and steady-state concentrations were obtained each time. The measured values were compared with the reference group without gastrectomy. The C_max_ of ciclosporin and the C_max_ and AUC of tacrolimus were higher than in the patients without gastrectomy. The patient 5 years after gastrectomy was characterized by good absorption of the drugs under study. The rate and extent of sirolimus and MPA absorption from EC-MPS was similar to the reference group. The MPA from MFF was absorbed worse than from EC-MPS [58].

A case of a patient who underwent liver transplantation and sleeve gastrectomy at the same time was also described. The patient received tacrolimus and everolimus. The appropriate level of immunosuppression was maintained. No drug absorption problems were observed. According to Tariciotti et al., it is beneficial for the patient to carry out these two treatments at the same time [64].

### 2.6. Thyroid Hormones

Gadiraju S. et al., conducted a meta-analysis of the effect of the type of bariatric surgery on the dosage of levothyroxine. Levothyroxine is a synthetic thyroid hormone used to treat diseases of the thyroid gland resulting from thyroxine deficiency. The drug is mainly absorbed in the jejunum and ileum. This fact suggests a higher demand for levothyroxine after jejunoileal bypass surgery. Although the jejunoileal segment of the small intestine remains intact for RYGB, SG and gastric banding, there are alternate variations to these procedures, which may result in an increased demand for levothyroxine. Probably, the small gastric sac in these procedures reduces the dissolution of levothyroxine in the stomach and results in an increased *t_max_* after RYGB. SG accelerates gastric emptying, which may contribute to the malabsorption of levothyroxine. However, most patients after RYGB and SG have a lower demand for levothyroxine. Presumably, this could be explained by the correlation between a change in the body weight and a change in levothyroxine dosage. In obesity, highly lipophilic drugs such as levothyroxine have an increased volume of distribution, which changes their pharmacokinetics. Probably, weight loss after bariatric surgery regulates the pharmacokinetics and results in a lower demand for levothyroxine. Obese patients have not only a greater mass of the adipose tissue but also a higher lean body mass, which is responsible for 20–40% of the increase in the total body weight. T4 is converted into T3 in the skeletal muscles, so it is likely that the reduction in the lean body mass after bariatric surgery results in a lower postoperative demand for levothyroxine. Lower serum leptin levels may also decrease the demand for levothyroxine in the postoperative period. Leptin regulates the expression of the thyrotropin-releasing hormone (TRH) gene and thus stimulates the production of TSH. The loss of weight causes the serum leptin and TSH levels to decrease and reduces the demand for levothyroxine. (SG (4 articles), RYGB (6 articles), biliopancreatic diversion (1 article), gastric banding 2 (article), and jejunoileal bypass (3 case reports)) [65].

### 2.7. Antidiabetic Drugs

More than 40% of patients with diabetes remission after gastric bypass surgery may redevelop diabetes within five years. Metformin is an oral antidiabetic, antihyperglycemic drug absorbed mainly in the upper part of the small intestine. When administered orally, it is characterized by low bioavailability of 29–60%. Padwal R. et al., studied changes in the pharmacokinetics of metformin on 16 non-diabetic post-gastric bypass patients. Surgical patients were examined ≥ 3 months after surgery. The control group consisted of 16 people selected in terms of sex and BMI (mean age 40 years and BMI 39.2 kg/m^2^). All of them were given two 500 mg metformin tablets and then their plasma levels were measured. In comparison with the control group, the metformin AUC_0–∞_ in the patients with a gastric bypass increased by 21% (13.7 vs. 11.4 μg/mL/h; *p* = 0.20), whereas bioavailability increased by 50% (41.8 vs. 27.8%; *p* = 0.007). The C_max_ in the group of patients was 2.0 mg/mL, whereas in the control group it was 1.8 mg/mL (*p* = 0.32). The results showed that the gastric bypass increased the metformin exposure, and this may cause the risk of toxicity. A change in the dosage of the drug should be considered [66]. However, Perrone et al., revealed that laparoscopic Roux-en-Y gastric bypass showed better effectiveness in type 2 diabetes mellitus resolution rate in comparison to laparoscopic sleeve gastrectomy [67].

### 2.8. Loop Diuretics

Furosemide is a loop diuretic drug. It is mainly absorbed in the stomach. Its peak diuretic effect occurs approximately one hour after oral administration. The bioavailability is extremely variable (10–90%). Furosemide is highly bound to plasma proteins (>95%). About 50% of furosemide is excreted in an unchanged form with urine, whereas the rest is metabolized to glucuronide by the kidneys. Patients with renal impairment exhibit a reduced response and a prolonged half-life of furosemide due to decreased urinary excretion [68]. Oral furosemide was administered to 13 RYGB patients and 14 healthy subjects (the authors do not specify the time of the examination after the surgery; the term “several” was used). The *t_max_* of furosemide was (1.8 ± 0.3 vs. 4.2 ± 1.2 h (*p* = 0.006). However, there were no differences between the groups in the six-hour urine volume. The maximum plasma concentration and half-life were not different, either [69].

### 2.9. Proton-Pump Inhibitors

Omeprazole is a proton-pump inhibitor that effectively suppresses the secretion of gastric acid in the parietal cells. The drug is formulated as encapsulated granules to prevent its degradation in an acidic environment. Although omeprazole is well absorbed from the gastrointestinal tract, its oral bioavailability in humans is about 40–50%, which suggests its pronounced first pass metabolism. Oral omeprazole was administered to 18 RYGB recipients and 18 healthy subjects (the authors do not specify the time of the examination after the surgery; the term “several” was used). In comparison with the control group, the *t_max_* of omeprazole in the RYGB group was significantly shorter (1.1 ± 1.1 vs. 4.4 ± 1.3 h, *p* < 0.0001). The maximum plasma concentration, half-life, area under the curve, and oral bioavailability were not different [69,70].

### 2.10. Vitamins

Vitamin D maintains calcium homeostasis and optimizes bone mineralization. Prolonged vitamin D deficiency leads to hypocalcemia, osteopenia, and osteoporosis. During the first month after RYGB surgery there are higher 25-hydroxyvitamin D concentrations, which decrease in the following months. These observations suggest increased storage and sequestration of vitamin D by the adipose tissue with its simultaneous release during the initial weight loss. After BPD/DS there is also a progressive increase in the incidence and severity of vitamin D deficiency. Due to the increased risk of metabolic bone disease in patients after bariatric surgery, lifetime prophylaxis consisting of oral vitamin D supplementation is recommended [71].

Vitamin B12 is responsible for the proper function of the nervous system. The development of vitamin B12 deficiency in patients after bariatric surgery is mainly caused by reduced production of the intrinsic factor by a limited number of parietal cells. In consequence, there is reduced formation and absorption of the cobalamin-intrinsic factor complex. Purely restrictive surgery does not result in significant deficiency of any of the nutrients. However, about a third of patients undergoing mixed procedures such as RYGB develop vitamin B12 deficiency [71]. A comparative study of RYGB and SG showed that the risk of vitamin B12 deficiency was 3.55-times higher after RYGB than after SG [72]. Similarly, folate deficiency commonly occurs after bariatric surgery—according to reports, its incidence after RYCB is 45%. Vitamin B12 and folic acid supplementation are recommended to patients after bariatric surgery [71].

Bariatric procedures and other mixed treatments may result in malabsorption of fats due to a biliary pancreatic lesion. In consequence, there is significant deficiency of fat-soluble vitamins. The researchers observed that one year after BPD/DS, 52% of the patients under study had vitamin A deficiency, whereas 51% had vitamin K deficiency. Pre- and postoperative fat-soluble vitamin assessment and routine supplementation are recommended [71].

### 2.11. Mineral Elements

Due to impaired nutrient absorption and/or reduced food intake, patients after bariatric surgery develop nutritional deficiencies, which may lead to anemia and osteoporosis. Anemia occurs much more often after RYGB (45–50%) than after SG (17%). In most cases it is caused by iron deficiency. This microelement is absorbed mainly in the duodenum and proximal jejunum. Iron deficiency is mainly caused by the bypass of these parts of the gastrointestinal tract and by hypochlorhydria [73]. Enani et al., observed an incidence of iron deficiency of 24.5% after RYGB and 12.4% after SG [74]. The American Society for Metabolic and Bariatric Surgery recommended iron supplementation to all bariatric patients. The effectiveness of iron supplementation can be increased by combining iron with vitamin C or citrus fruit [75].

Reduced bone mineral density and increased bone turnover are some of the consequences of dietary restrictions and bariatric surgery. Researchers observed that the bone loss after RYGB was greater than after SG. The bone loss is mainly caused by calcium deficiency, which affects almost 10% of bariatric patients. These patients exhibit reduced calcium absorption because the main sites of absorption of this element (the duodenum and proximal jejunum) are bypassed [73]. Schafer et al., conducted a study to determine the effect of Roux-en-Y gastric bypass surgery on intestinal fractional Ca absorption (FCA) (*n* = 33). Despite the recommended daily calcium intake of 1200 mg, the FCA was significantly lower after RYGB. Before the surgery it was 32.07%, whereas six months after the surgery it dropped to 6.9%. Researchers suggest that bariatric patients may require a higher dose of calcium than recommended [76].

## 3. Conclusions

Table 2 lists changes in the pharmacokinetic parameters observed after bariatric surgery and gastrectomy.

## Figures and Tables

**Table 1:** Stages for including articles into the review.

Stage	Action	Results
I	Key words (‘gastrectomy’, ‘gastric bypass’, ‘bariatric surgery’ AND ‘pharmacokinetic’, ‚absorption’ and ‚bioavailability) Databases: Cochrane, PubMed, Scopus	814
II	Due to the small number of studies in recent years, the time criterion has not been applied	383
III	Clinical trial	383
IV	Independent verification and inclusion of research by two authors inclusion criteria: The groups of drugs selected by the authors: Antibiotics, Analgesic drugs, Antidepressants, Anticoagulant drugs, Immunosuppressants, Thyroid hormones, Antidiabetic drugs, Loop diuretics, Proton-pump inhibitors, Vitamins, Mineral elements	34

**Table 2:** Changes in the pharmacokinetic parameters observed after bariatric surgery and gastrectomy.

Drug	Type of Surgery	Time after Surgery	Study Group Size	Changes in PK/PD	References
Acetaminophen	SG	4–6 months	9	↑AUC, C_max_ and *t_max_*	[17]
	RYGB	6–7 days	30	↓C_max_ and AUC	[34]
Tramadol	RYGB	7–10 days	26	↓C_max_ (eff) ↓*t_max_*	[35]
Ketoprofen	total gastrectomy	6–11 days	15	↓C_max_ and *t_max_* ↑V_d_	[6]
	partial gastrectomy	6–11 days	5		
Morphine	RYGB	6 months	30	↑AUC, C_max_ ↓*t_max_*	[39]
Oxycodone	total gastrectomy		24	↓*t_max_*	[40]
Rivaroxaban	SG	3 days	6	↑AUC and C_max_	[49]
	RYGB	3 days	6	↑AUC and *t_max_* ↓C_max_	
	SG	6–8 months	6	↑*t_max_*	[50]
	RYGB	6–8 months	6	↑*t_max_* ↓C_max_	
Dabigatran	RYGB		9	↓C_max_	[52]
Warfarin	RYGB	6 months	12	Maintaining the INR level required lower doses	[56]
Tacrolimus	SG	9–12 months	12	↑AUC and C_max_	[63]
	RYGB		6	↓AUC:dose ratio	[61]
	RYGB		1	↑C_max_ and AUC	[58]
Mycophenolate mofetil	SG	9–12 months	12	↓Cl/F ↑*t*_1/2_	[63]
Enteric-coated mycophenolate sodium	SG	9–12 months	12	↓Cl/F ↓*t_max_*	[63]
Sirolimus	RYGB		6	↓AUC: dose ratio ↓AUC_0–∞_ and AUC_0–12_	[61]
MPA (active form of mycophenolate mofetil)	RYGB		6	↓C_max_ and mean AUC_0–12_	[61]
Ciclosporin	RYGB		1	↓C_max_	[58]
Escitalopram	RYGB	2 and 6 weeks	4	↓C	[42]
Sertraline	RYGB	9–15 months	5	↓AUC_0–10.5_ and C_max_	[43]
Duloxetine	RYGB	9–15 months	10	↓AUC_0–∞_ and t_max_	[45]
Vortioxetine	RYGB	91 days	1	↓C	[48]
Metformin	RYGB	≥3 months	16	↑AUC_0–∞_ and C_max_	[66]
Furosemide	RYGB	<12 months	13	↑*t_max_*	[69]
Omeprazole	RYGB	<12 months	18	↓*t_max_*	[69]
Levothyroxine	meta-analysis				[65]

SG—sleeve gastrectomy. RYGB—Roux-en-Y gastric bypass. AUC—area under the curve. AUC_0–∞_—area under the curve from 0 to infinity. C_max_—maximum concentration. *t_max_*—time of maximum concentration. Cl/F—clearance. V_d_—volume of distribution.

## Data Availability

Not applicable.
